# Validation of an Automated Screening System for Diabetic Retinopathy Operating under Real Clinical Conditions

**DOI:** 10.3390/jcm11010014

**Published:** 2021-12-21

**Authors:** Soledad Jimenez-Carmona, Pedro Alemany-Marquez, Pablo Alvarez-Ramos, Eduardo Mayoral, Manuel Aguilar-Diosdado

**Affiliations:** 1 Ophthalmology Department, Hospital Universitario Puerta del Mar, University of Cadiz, 11009 Cadiz, Spain; pablo.alvarez.ramos@gmail.com; 2 Comprehensive Healthcare Plan for Diabetes, Regional Ministry of Health and Families of Andalusia, Government of Andalusia, 41020 Seville, Spain; eduardo.mayoral.sspa@juntadeandalucia.es; 3 Biomedical Research and Innovation Institute of Cadiz (INiBICA), 11009 Cadiz, Spain; manuel.aguilar.sspa@juntadeandalucia.es

**Keywords:** diabetic retinopathy, teleophthalmology, diagnostic accuracy, population-based screening, sight-threatening diabetic retinopathy

## Abstract

Background. Retinopathy is the most common microvascular complication of diabetes mellitus. It is the leading cause of blindness among working-aged people in developed countries. The use of telemedicine in the screening system has enabled the application of large-scale population-based programs for early retinopathy detection in diabetic patients. However, the need to support ophthalmologists with other trained personnel remains a barrier to broadening its implementation. Methods. Automatic diagnosis of diabetic retinopathy was carried out through the analysis of retinal photographs using the 2iRetinex software. We compared the categorical diagnoses of absence/presence of retinopathy issued by family physicians (PCP) with the same categories provided by the algorithm (ALG). The agreed diagnosis of three specialist ophthalmologists is used as the reference standard (OPH). Results. There were 653 of 3520 patients diagnosed with diabetic retinopathy (DR). Diabetic retinopathy threatening to vision (STDR) was found in 82 patients (2.3%). Diagnostic sensitivity for STDR was 94% (ALG) and 95% (PCP). No patient with proliferating or severe DR was misdiagnosed in both strategies. The k-value of the agreement between the ALG and OPH was 0.5462, while between PCP and OPH was 0.5251 (*p* = 0.4291). Conclusions. The diagnostic capacity of 2iRetinex operating under normal clinical conditions is comparable to screening physicians.

## 1. Introduction

In 2019, the IDF (International Diabetes Federation) estimated that 463 million adults worldwide suffered from diabetes, and projected the number to rise to 700 million by 2045 [1]. The prevalence of diabetes in Andalusia, the most populated autonomous community in the south of Spain, is higher (15.3%) than in the rest of Spain (12.5%), in close relation to lifestyle and socioeconomic factors [2].

Retinopathy is the most common microvascular complication in patients with diabetes mellitus [3]. In developed countries, diabetic retinopathy (DR) is one of the leading causes of blindness among people of working age [4]. A recent meta-analysis has calculated that in diabetic patients aged 20−79 years, the overall prevalence of any DR is 35% [5]. While the global prevalence of DR and Diabetic Macular Edema (DME; potential complication of DR), for the period 2015 to 2019 were 27.0%, and this prevalence in Europe was estimated to be 20.6%, calculated from the results of population-based studies with retinography [6]. It has been known for decades that proper treatment of DR decreases the incidence of severe visual loss when early diagnosed [7]. Telemedicine systems enable the remote analysis of digital fundus photographs, thus detecting the presence of DR lesions. Based on this technology, population-based screening programs have been developed in different countries [8]. The growing number of diabetic patients and their periodic medical monitoring entails an increase of these DR-detection digital analyses. Due to the limited number of ophthalmologists, other professionals are required to address DR screening. In particular, these range from family physicians, endocrinologists or nurses in high-income countries to trained non-medical personnel in middle-income countries [9,10,11,12,13].

In recent years, the research effort has focused on the development of automated diagnostic strategies that can complement or replace screening personnel, which would help reduce the workload and improve access for diabetic patients requiring early diagnosis [14,15].

The Andalusian Public Health System (APHS) provides universal health care to the 8.4 million inhabitants of Andalusia, which represent 18% of the Spanish population. The APHS encompasses an extensive network, with two levels of care (1500 primary healthcare centers and 49 hospitals) based on accessible, high-quality, patient-centered care, in a system with universal coverage and funded by taxation. The APHS, through the program for Early Detection of Diabetic Retinopathy (APDR), which is part of the Comprehensive Healthcare Plan for Diabetes (CHPD), provides a network of digital desktop fundus cameras placed in primary healthcare centers throughout the region. The screening system developed by APHS consists of two phases: in the first phase, digital fundus photographs of diabetic subjects with no previous diagnosis of DR are recorded with a non-mydriatic retinal camera (NMRC). Secondly, the primary care physicians (PCP) of each center assess these photographs, and then, both those displaying a probable DR diagnosis and inconclusive ones are sent via a specific intranet to a reference ophthalmologist for diagnostic confirmation. From January 2005 to June 2019, 888,318 examinations were performed, corresponding to 429,791 patients [2]. It should be noted that patients with a healthy retina are examined periodically, which increases the number of images under study every year.

To reduce this increasing workload of PCP performing the screening activity, the Lynch Diagnostics (Granada, Spain) (LD) computer-aided diagnostic platform, in collaboration with APHS, developed the 2iRetinex algorithm. The 2iRetinex software (Granada, Spain) lassifies the screening images according to their quality. Specifically, for the non-rejected images, it provides information on the number, type and location of the lesions and, ultimately, a final diagnostic decision.

The aim of this research study is the validation of the 2iRetinex software as a complement or substitute for the screening physician. The clinical diagnosis of the screening physician is formulated in terms of the absence or presence of DR. To perform the comparison of this categorical diagnostic strategy with the same categories provided by the algorithm, we introduced the algorithm into the APDR system. The algorithm extracted the diagnosis of the presence or absence of diabetic retinopathy from the system, obviating the additional information. The agreed diagnosis of three specialist ophthalmologists is used as the reference standard (“ground truth”) to measure its clinical performance.

## 2. Materials and Methods

### 2.1. Study Participants from APDR

An analytical study of diagnostic tests was performed on the fundus images of diabetic patients regularly attending the Andalusian program for early diagnosis of diabetic retinopathy (APDR) circuit. The used protocols were approved by the Ethical Committee for Research and Clinical Trial of Hospital Puerta del Mar (Cadiz, Spain), number 62/16.

To compare the diagnostic capability of the 2iRetinex algorithm (ALG) with that of the screening PCP under usual clinical conditions, we calculated the required sample based on a previous APDR study with a prevalence of retinopathy in diabetic patients of 30% [16]. To obtain an average sensitivity of 85% and specificity of 82% for the screening PCP [16] and a sensitivity of 90% and a specificity of 93% for the ALG (internal unpublished data from the company, on a random sample of 575 patients in 2015), as well as, a type 1 and 2 error at 0.5 and at 0.2, respectively, the sample size estimated was 2421 patients. For a paired sample, the required sample size calculation was 2297 patients. Furthermore, as the rate of ungradable (UNG) images was estimated to be 10%, an additional 242 and 230 patients were added, for independent and paired samples, respectively [16].

Retinographies were obtained from all diabetic patients attending ten primary care health centers in the region of Andalusia (Spain) included in the APDR patient flow circuit using a standardized protocol previously described [16,17,18]. Briefly, three photographs of each eye were recorded using a retinal camera. The following camera models were used: the Topcon NW 200 (Topcon, Tokyo, Japan) in seven of the ten study centers and in the remaining three centers, the Topcon NW100 (Topcon, Tokyo, Japan), the Zeiss VISUCAM (Carl Zeiss Meditec AG, Jena, Germany) and DRS retinography, respectively. Then, the PCP analyzed the images stored on the APDR server. The images of the patients classified as UNG and those that present findings of DR are assigned, through the intranet, to the reference ophthalmologist who issued a clinical judgment of confirmation. If no DR lesions were detected (NODR) the patient continued in the screening system for future examinations.

### 2.2. 2iRetinex Software

**Figure 1:**
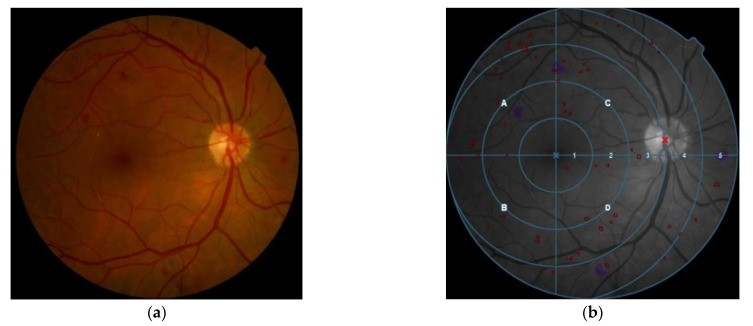
Software analysis 2iRetinex: (**a**) A original APDR image of fundus photography of a patient with diabetic retinopathy; (**b**) Location and type of lesions. Superimposed the coordinates system. Red lesions in red, white lesions in blue.

The 2iRetinex software (patent number RPI201499900601833) extracted different features from the retinographies. Specifically, 2iRetinex extracted the vascular tree through the isolation of the green channel and microaneurysms and hemorrhages (which are candidates for lesions by applying structural characteristics), through analysis of the dark residual objects. In addition, the 2iRetinex software superimposed a binary mask on the green layer of the images, which made it possible to locate the optic disc. Using a filter also highlighted the bright lesions (exudates and cotton spots). Furthermore, it was able to detect the position of the macula. Finally, a topographic reference system was established to locate the lesions by coordinates. After checking that the image was suitable for analysis, the original image (Figure 1a) was transformed into the resulting image shown in Figure 1b.

### 2.3. Source of Images and Data

In the participating centers of this study, between April 2017 and June 2018, a capture system was installed between the retinography and the APDR network. This system allowed the original images in TIFF format to be sent to the LD diagnostic platform to obtain the patient’s clinical diagnosis through the 2iRetinex software (ALG) using the same diagnostic categories as the PCP. These images, but not the diagnoses, were available on a server for the ophthalmic researchers (SJC, PAM, PAD). The latter were the ones who established the reference diagnosis (OPH). Simultaneously, the images were returned to the APDR network for compression into JPG for final screening by the PCP, following the usual circuit. The PCPs were not aware of the parallel diagnostic systems. Moreover, at no time were the real graders aware of the diagnosis issued by the other study participants before issuing their clinical judgment. 

To ensure that the study was double-blind, the LD platform provided an internal control code. Once the OPH and ALG diagnoses were established, the list of codes related to the original health identification was sent to the administrative management of the APDR. Likewise, the demographic data of the study sample and the PCP diagnoses were sent from the APDR for cross-checking. 

For each patient, data were obtained for the categorical variables (gender and type of diabetes) and the quantitative variables (age and years of duration of the disease at the time the retinographies were taken; years from diagnosis). The database also recorded the use of pre-exploration mydriatics. 

### 2.4. Diagnostic Criteria and Convention

For each patient, the PCP, ALG and OPH diagnosis were obtained, with the same diagnostic categories. The following criteria were used to classify the images: 

If the image lacked sufficient quality to confirm or rule out diabetic lesions, it was classified as Ungradable (UNG). 

If the image quality was sufficient and no diabetic retinopathy lesions were found, it was classified as No Diabetic Retinopathy (NODR) 

Regardless of the quality of the image, if retinopathy lesions were observed, the image was classified as DR.

For the final diagnosis of each patient, as carried out in the APDR circuit, the following criteria were followed: 

If at least one of the patient’s two eyes was classified as UNG, the patient was diagnosed as UNG.

If DR lesions were detected in the images of at least one eye, the patient was diagnosed as DR. 

If none of the images of the patients considered evaluable showed lesions of DR, the patient was diagnosed as NODR. 

Research ophthalmologists (SJC, PAM, PAD) classified the stage of DR according to the International Clinical Diabetic Retinopathy Severity Scale [18]. This scale consists of five stages: no diabetic retinopathy (NODR), mild (MILD), moderate (MOD), and severe non-proliferative DR (SEV), and proliferative retinopathy (PROL). The diagnosis of diabetic macular edema (DME) was established by detecting the presence of any signs suggestive or evident of macular edema in at least one eye. Cases diagnosed as DME, SEV and PROL were considered as patients with sight-threatening DR lesions (STDR). If the patient had DR lesions in both eyes, the stage of diagnosis corresponded to that of the eye with the highest degree of DR.

### 2.5. Statistical Analyses

The study data were analyzed using descriptive statistics. Means and standard deviations were calculated for quantitative variables and proportions for qualitive ones. We used the Chi-squared test and the t-test to compare proportions and means, respectively. Sensitivity, specificity, predictive values, and likelihood ratios were used to assess the accuracy of diagnostic tests. Likewise, reliability and agreement were quantified using kappa coefficient. The kappa concordance values of each test (ALG and PCP) were established with respect to the OPH diagnosis and the similarity of the results were examined. The area of the simple ROC curve was calculated for each examiner with respect to ground truth and compared between them. Level of significance was estimated at *p* < 0.05.

Statistical analyzes performed using IBM SPSS Statistic v 24 software (Armonk, NY, USA). The sample calculation and the capacity of the diagnostic tests have been quantified using the Epidat 3.1 program (Xunta de Galicia, Galicia, Spain) (www.sergas.es/Saude-publica/EPIDAT).

## 3. Results

### 3.1. Analysis of the Original Sample. Gradable/Ungradable Concordance

During the download period, image folders of 3575 diabetic patients were obtained from the 10 primary healthcare centers. Due to duplication of files and empty downloads without files, 55 patients were removed. Therefore, the original sample consisted of 3520 image folders, of which 43,9% corresponded to women (Table 1). The average age of the patients was 64.4 ± 14.6 years and the mean of the years since the diagnosis of diabetes was 10.37 ± 7.5 years. The majority of patients (88.2%) had type 2 diabetes mellitus (DM2), while patients with type 1 diabetes mellitus (DM1) accounted for 11.4%. In contrast, a minimum proportion (0.4%) of the sample corresponded to patients either diagnosed with other categories or without detailed diagnosis (Others). 

Six hundred and fifty-three patients (18,5%) had images with features of DR. Macular edema was detected in 77 patients (2.2%) and DR stage became STDR in 82 patients (2.3%). However, it was not possible to establish diagnostic criteria on the condition of the retina in 11.6% of the images (UNG).

Regarding the distribution by gender, the mean age and mean duration of diabetes were significantly higher among the diabetic females. In addition, in female patients, the proportion of DM1 was higher, and the proportion of DM2 was lower than in the male group. However, there was no significant difference in the prevalence of each stage of DR between genders (Table 2).

Considering the gradable/non-gradable (GRAD/UNG) category of images (previously described in methods), we found that the group of patients with UNG images showed significant differences in the mean age and proportions of the types of diabetes compared to patients with GRAD images (Table 3). Specifically, our data showed that patients with UNG images were 9.3 years older than patients with GRAD images (72. 6 vs. 63. 4). We also found a statistically significantly higher proportion of type 2 diabetic patients and a lower proportion of type 1 diabetic patients, in the group of patients with UNG images (Table 3).

Regarding the graders used, the images of 407 patients (11.6%) were classified as UNG according to the consensus diagnosis of the OPHs (Table 4 and Table 5). Likewise, the automatic analysis software considered the images of 927 patients (26.3%) as inadequate (Table 4), while the PCPs could not provide any diagnosis in 461 patients (13.1%; Table 5). A significant difference was detected in the proportions of UNG images between ALG and OPH classifier strategies and between ALG and PCP classifier strategies (*p* < 0.0001). The difference of 1.54% in the proportions of UNG images between OPH and PCP also reached the limit of significance (*p* = 0.0494).

The unweighted Kappa statistic was used to assess inter-rater reliability in classifying images into UNG or GRAD. The Kappa statistic shows the observed level of agreement adjusted for the level of agreement that could have occurred by simple chance. A value of 0.75–1.00 indicates an excellent agreement, 0.4–0.75 represents a moderate agreement, and lower values display the deficient agreement. We found that the kappa value was 0.3623 for the agreement between ALG and OPH classifier strategies. The agreement between PCP and OPH obtained a k-value of 0.3144. The kappa homogeneity test showed a Chi-square value of 2.7274 corresponding to a *p* = 0.0986. Therefore, the difference in agreement between the two diagnostic strategies was not statistically significant.

Description of the distribution of the diagnostic categories UNG, DR and NODR of the two diagnostic strategies compared to the criteria of the ophthalmologists:

The proportions of the GRAD/GRAD and UNG/UNG agreement categories for ALG versus OPH were 0.78 and 0.30, respectively, with a composite agreement ratio of 0.80. The proportions of the GRAD/GRAD and UNG/UNG agreement categories for PCP versus OPH were 0.84 and 0.25, respectively, with a composite ratio of 0.85. 

Overall, these results demonstrated the proportion of UNG was higher in ALG, and the proportion of composite agreement was higher in PCP. It should be noted that the concordance between ALG and OPH was slightly better (0.3623 vs. 0.3144) because there was a greater coincidence when classifying UNG images (ALG 0.30 vs. PCP 0.25). 

### 3.2. Comparison of DR/NODR Diagnostic Category in Unpaired Samples. Diagnostic Validity

The 407 patients considered as UNG by the OPHs were removed from the original sample. This new sample of 3113 patients was partially described when the comparison between GRAD vs. UNG samples was carried out (Table 3). Based on the results of Table 4 and Table 5, two samples were selected. The first sample, called ALG-OPH, contained all patients considered GRAD by these two graders (*n* = 2496). The second sample, called PCP-OPH, included all patients considered GRAD by these other pairs of graders (*n* = 2825).

Our data showed that the PCP-OPH group had a mean age of 1.4 years, significantly higher than that of the ALG-OPH group (*p* = 0.0003). No significant differences were detected between the two samples for any of the other variables (Table 6).

The frequencies of the matching of the diagnostic categories of each strategy with the criteria of the OPH and the values of indexes (sensitivity, specificity, positive and negative predictive values, likelihood ratios) that determine the validity of the diagnostic tests with the different classifier strategies are shown in Table 7. In particular, the ALG strategy showed a greater ability than the PCP to detect retinopathy in diabetic patients’ retinographies (Sensitivity, 85.05 vs. 64.54). However, the PCP strategy better identified individuals without retinopathy as demonstrated by its specificity values (80.67 vs. 89.59). These results suggest that the diagnostic ability of the ALG was overall superior to that of the PCP.

Four of the 80 DR patients classified as healthy by the ALG were positive for MOD plus DME and in the case of the PCP strategy it was 4 out of 214. The proportion of STDR patients incorrectly diagnosed was 5.9% (4/67) for the ALG and 5.3% (4/75) for the PCP. There was no statistically significant difference in the proportions of misdiagnosed patients between the two strategies (ALG vs. PCP; *p* = 0.8693). In fact, the sensitivity of the ALG strategy to diagnose STDR was 94% and that of PCP 95%. No patient with severe or proliferative retinopathy was misdiagnosed by ALG or PCP. Both strategies had a low positive predictive value (PPV; Table 7), being even lower for ALG (PPV 54.56 vs. 61.55) but a high negative predictive value (ALG 95.19 vs. PCP 90.36). The ALG strategy not only had a higher sensitivity, but also a better negative predictive value than the PCP strategy.

Likelihood ratios (LR) are another alternative for calculating the diagnostic accuracy and summarizing the information endowed in both sensitivity and specificity. The LR is the probability that a particular test result would be expected among patients with the condition diagnosed compared to the likelihood that that same result would be expected in a patient without the condition. Good diagnostic tests have LR+ > 10 and their positive result has a significant input to the diagnosis. Good diagnostic tests have LR- < 0,1. The lower the LR- the more significant contribution of the test is in ruling-out (Table 8)

In the ALG strategy, the modification of the previous probability for positive test results was small (LH + 4.40, Table 7). In fact, we calculated the post-test probability using Fagan’s nomogram and obtained that the probability of a patient having a DR changed from 21% to 55%. About 1 in 1.8 positive tests corresponded to patients with retinopathy. The subsequent probability, if the test was negative, was modified to 5%, so approximately 1 of each negative result corresponded to an individual without DR. Likewise, the modification of the previous probability in the PCP test, for positive results, was moderate (LH + 6.10), changing the probability of a patient having a DR from 21% to 62% (Fagan’s nomogram). About 1 in 1.6 positive tests corresponded to patients with retinopathy. The subsequent probability, if the test is negative, was modified from 21% to 10%, so approximately 1 in 1.1 negative results corresponded to an individual without retinopathy. 

**Figure 2:**
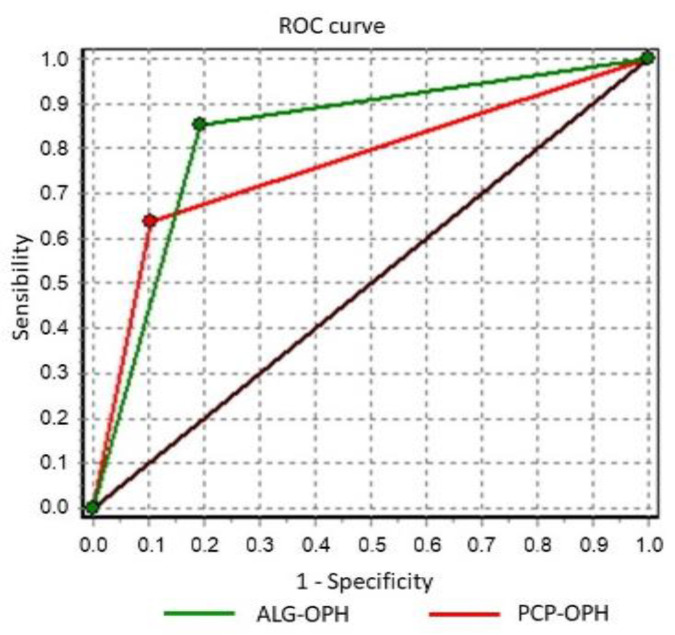
ROC curves comparison. Green line: Algorithm. Red line: Primary Care Physician.

To determine the discriminative power of the different classifier strategies of DR diagnosis, our results were compared using the area under the curve (AUC) of the Receiver Operating Characteristics (ROC) curve (Figure 2).

The AUC for the PCP displayed a value of 0.7657 (95% CI 0.7452 to 0.7861), while the value of AUC for ALG was 0.8286 (95% CI 0.8111 to 0.8461). We found that there was a significant difference between the two curves (*p* = 0.0000). Therefore, these power results shown in Figure 2 indicate that the ALG was more efficient than the PCP.

Finally, to assess the intergrader reliability for DR diagnosis, we used the Kappa statistic. We found that the k-value of the agreement between the ALG and OPH was 0.5462 (95% CI 0.5109 to 0.5815), while between PCP and OPH was 0.5251 (95% CI 0.4865 to 0.5636). The kappa homogeneity test showed a Chi-square value of 0.6252 corresponding to a *p* = 0.4291. Therefore, the difference in concordance between the two diagnostic strategies was not statistically significant. 

### 3.3. Comparison of Diagnostic Tests in Paired Samples

To confirm the previous results, we studied the patients classified as GRAD by the three diagnostic strategies, removing from the original sample all patients considered UNG for any of the strategies. In Table 9, the proportions and means of the paired sample obtained were described. 

The frequencies of pairs of diagnostic categories of each strategy compared to the criteria of the OPH, and the values of the diagnostic validity indexes for the sample described are displayed in Table 10.

The pattern of the indexes shown in Table 10 is comparable to that found in the unpaired sample (Table 7). In fact, the prevalence of DR was the same in all the samples. In summary in the paired sample, the ALG showed a greater ability than PCP to detect DR lesions. However, PCP better identified patients without DR.

Similarly, as in the unpaired sample, we determined that the probability that an individual with a positive test would have retinopathy was small in both strategies (PPV 54.36% vs. 60%), being even lower for the ALG. Both tests presented a high negative predictive value. Therefore, for the unpaired sample, we can conclude that the 2iRetinex software is a good predictor of the absence of DR and a moderate predictor of the presence of DR. In contrast, the diagnostic utility of PCP screening is considered good for positive results and fair for negative.

**Figure 3:**
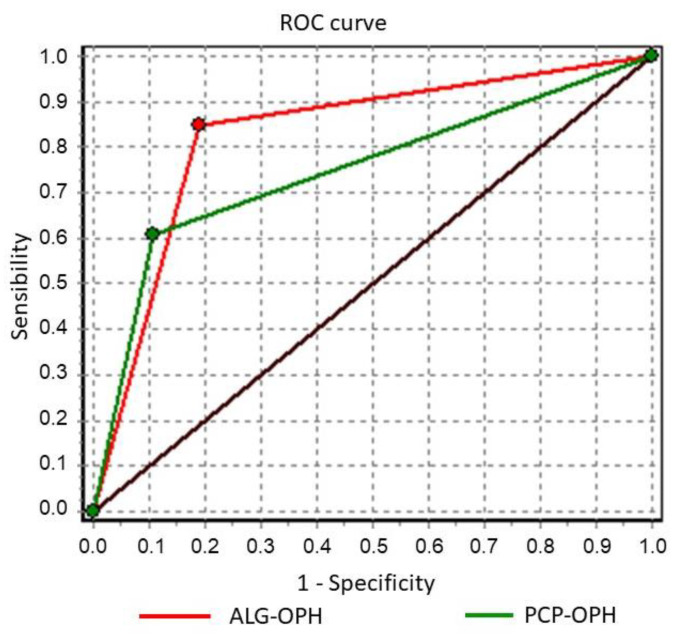
ROC curves comparison of the diagnostic validity. Paired sample. Red: Algorithm. Green: Primary Care Physician.

The AUC for the PCP showed a value of 0.75, while for the ALG was 0.8287. We found that there was a significant difference between the two curves (Chi-square value 28.0575, *p* = 0.0000). Consequently, these results shown in Figure 3 indicate a greater diagnostic power of the ALG.

The k-value of the concordance between the ALG and OPH was 0.5455, while between PCP and OPH it was 0.4974. The homogeneity test for the difference in k-values was not significant (Chi-square 2.7898 *p* = 0.949). 

## 4. Discussion

Digital fundus photography is considered an acceptably accurate procedure for detecting DR [13]. Image processing and interpretation in DR screening programs require initial training and ongoing updating for all personnel involved. This entails personal effort and resource consumption. Population-based screening programs for detecting DR help reduce visual loss by identifying sight-threatening cases and referring them to specialists for treatment. A recent meta-analysis of 33 studies worldwide concludes that teleophthalmology has a moderate sensitivity and high specificity for detecting the absence of DR. However, the results obtained for the diagnosis of the diseased retina show widespread variations [19]. In recent years, there has been increasing interest in applying automation processes in ophthalmic telemedicine to reduce the need for screening professionals and to homogenize diagnostic criteria regardless of the origin and composition of the sample being evaluated [20]. For that reason, in this study, we assessed the use of the 2iRetinex software as a complement or substitute for the screening physician. 

In this assessment of images from 3520 patients from ten APDR primary care centers, the prevalence of any form of DR was 18.5%, while STDR cases accounted for 2.3% of the study subjects. Similar results were obtained in the first-year screening of the Scottish program, where the prevalence of DR and STDR was 19.3% and 1.9%, respectively. These values increased in the subjects reviewed one year (20.5% and 2.3%, respectively) [21]. Likewise, other studies with similar characteristics showed a percentage of STDR ranging from 2.57% to 4% [8,22,23].

According to the American Telemedicine Association Validation Level, the first phase of our APDR screening system can be classified as a category 1 program, with an on-disease/non-disease diagnostic criterion issued by the PCP [24]. Meanwhile, the second phase, characterized by the review of pathological retinographies by the referring ophthalmologist is classified as a category C2 program. The ophthalmologist establishes the stage and the time frame for the patient’s review or for initiating treatment. Previous studies indicated that artifacts may be present in 3−30% of the photographs without mydriasis [25]. Of note, widespread use of tropicamide (97.8% of patients) does not pose any additional risk [26]. In this regard, the proportion of non-valuable retinal images in published studies is highly dependent on the age distribution of the sample, which in turn is related to the presence of eyelid and corneal abnormalities and especially cataracts. In our study, despite the use of tropicamide to dilate the pupil, the proportion of patients with UNG images was 11. 6%. Moreover, PCPs were unable to make a judgement on 13. 1% of patients. Likewise, the automatic analysis of the algorithm considered 26.3% to be unclassifiable. Our data are consistent with those of other studies. In particular, in a validity study, the iGrading automatic assessment system found 26.16% of 2309 patients to be ungradable [27]. The Italian multicenter study NO BLIND classified 23.4% of the telediagnostic images as “poor quality non-diagnostic images” [28]. Following this line in another study, the ratio of non-gradable patients classified by the automatic system was twice that of the human classification [22]. Indeed, in this study, 404 patients by the criteria of the automatic system were discarded, and 197 patients by the criteria of the ophthalmologist. Only in two other cases did they agree in classifying them as non-gradable [22]. Similarly, in our study, we found the same lack of diagnostic agreement in image grading between ALG and PCP with respect to OPH (k = 0.3623 and 0.3144, respectively). This suggests that the mechanisms underlying decision-making are different in algorithms and clinicians. The grading criteria of the automatic systems may have been more demanding than those of the humans, but what is evident is that their implementation is invariable and not affected by the inherent inconsistency of human subjectivity [29].

Our results on the diagnostic accuracy of screening PCP for any grade of DR showed a good mean specificity (89.6%) and a reduced sensitivity (63.5%). However, for STDR the sensitivity increased to 95%. These values are similar to those reported in other studies [8,11,26,30]. Noteworthy, our findings are consistent with a previous partial study about the diagnostic ability of screening PCP at three APDR primary care centers in a small sample of patients [17]. Similarly, the concordance in the diagnosis of DR with respect to the clinical judgment of the ophthalmologist was k = 0.408 and sensitivity was 97% and specificity 80%. 

The second level of APDR development, which would make it a screening and follow-up program to monitor patients with mild and moderate retinopathy without macular edema within the program, has not yet been implemented [30]. It could be that the screening physicians want to reduce the workload of ophthalmologists at the second and third levels of care by under-diagnosing cases that do not require referral. 

Regarding the results related to the diagnostic accuracy of the 2iRetinex software analysis test (ALG), an overall sensitivity of 85% was obtained for any degree of retinopathy and a specificity of 81%. For STDR cases, sensitivity increased to 94%. It should be noted that the agreement with the diagnosis of the reference ophthalmologists was similar to that of PCP and the area of the simple ROC curve (0. 8286) was slightly similar to that of PCP. Cost-effective ALG screening programs to identify DR lesions on digital images have been reported previously [31]. In particular, Fleming et al. [31] stated that this inclusion of algorithms increased the sensitivity to 100% in DR detection and reduced manual screening by more than 35%. 

EyeArt, Retmaker, iGrading or IDx are commercially available systems that have been used in teleophthalmic screening programs for DR diagnosis [20]. The functionality of iGrading is comparable to that of 2iRetinex by combining an image quality system and a DR identification criterion. In the validation study conducted in Valencia [23], iGrading showed excellent sensitivity values of 97.4% and specificity values of 98.3% for patients with STDR. For its part, iGrading has been used as a level 1 grading in the Scottish screening program after extensive validation since 2010 with a sensitivity of 97.8% and specificity of 41.2% for referable DR [32]. The other two systems, Eyeart and Retmaker showed good diagnostic accuracy with a sensitivity for STDR of 94.7% and 85%, respectively [33]. These methods qualified as Automated Retinal Image Analysis System (ARIAS) have not yet developed a sufficient level of autonomy to establish a classification of the patient’s damage and recommend treatment [34].

ARIAS have been changed with the development of artificial intelligence systems and Deep learning (DL), a subtype of machine learning (ML) that does not require image engineering. Early ML techniques for detecting DR used mathematical image transformation techniques and image engineering [35]. Moreover, DL develops its own pattern recognition representations after being fed raw data [36,37].

In relation to this, it is known that IDx has updated its ARIAS with an artificial intelligence system. The new version IDx-DR v2 is designed to identify DR referable to a specialist without human supervision. IDx-DR v2 achieved 100% sensitivity and 81.82% specificity for derivable DR and 100% sensitivity and 94.64% specificity for STDR in the sample examined [38]. A meta-analysis demonstrated that ML algorithms have a high diagnostic accuracy for the diagnosis of DR on color fundus photographs suggesting that they may also be ready for clinical application in screening programs [29]. However, early results published in relation to this ML had methodological inconsistencies, such as lack of external validation and the presence of biases. Most of these methods do not provide full interpretations of the relevant findings of retinal pathological signs. Furthermore, it should be noted that, at present, the full implementation of this “black box classification system” presents difficulties for acceptance by clinicians and patients [39].

The main limitation of our study is the dichotomous diagnosis made by the screening PCP. At least at this stage, the APDR screening program is not designed for PCP to classify the DR stage. Indeed, this limitation has prevented us from obtaining full diagnostic validity indexes results in patients with STDR.

The diagnostic capability for STDR stages is excellent for both diagnostic strategies (PCP and ALG). In the case of the overall diagnostic ability for any form of diabetic retinopathy with our algorithm, the 2iRetinex and PCP strategies are comparable. Moreover, their agreement with respect to the re-evaluation of ophthalmologists does not show significant differences. Therefore, the introduction of this real-time software into the APDR workflow would allow knowing whether the images taken of the patient’s fundus are of sufficient quality before the patient leaves the examination center. Thus, a re-examination could be recommended if necessary. This strategy would reduce the delay of repeat examinations and consequently, less disrupt the patient’s social and work activity. All this suggests that the automatic examination system could be introduced in a real way by integrating it with the screening physician in the first phase. 

In this study, we have validated our DR prediction software (2iRetinex) on a sample of patients under real clinical conditions in the routine APDR circuit. Although further assessment is needed to validate the system, it has been confirmed as a tool that could be integrated into DR screening programs. This could improve the quality of screening models in the future. Due to this, studies of the combined use of algorithms and manual classification emerge as an urgent need to achieve better performance. Thereby, the workload of manual classification could be minimized. We plan to conduct studies that focus on extending the algorithm using the 2iRetinex software to detect other common comorbid eye diseases such as age-related macular degeneration (AMD) and glaucoma.

## Figures and Tables

**Table 1:** Main characteristics of the study sample obtained from de APDR between April 2017 and June 2018. * Mean SD. DR = diabetic retinopathy. DME = diabetic macular edema. NDME = no diabetic macular edema. STDR = sight-threatening DR.

Variable of Interest			Study Sample (*n* = 3520)	
Gender (F/M)			43.9%/56.1% (1545/1975)	
Age (years)			64.4 ± 14.6 *	
Years from diagnosis			10.4 ± 7.5 *	
Mydriasis (before photograph)			97.8%	(3443)
Diabetes type	Type 2		88.2%	(3103)
	Type 1		11.4%	(402)
	Others		0.4%	(15)
Ungradable images (UNG)			11.6%	(407)
No diabetic Retinopathy (NODR)			69.9%	(2460)
Diabetic retinopathy (DR)	Mild		8.1%	(286)
	Moderate	NDME	8.1%	(285)
		DME	1.8%	(65)
	Severe	NDME	0.1%	(2)
		DME	0.2%	(8)
	Proliferative	NDME	0.1%	(3)
		DME	0.1%	(4)
STDR			2.3%	(82)

**Table 2:** Comparison of the study variables between female and male patients. DR = diabetic retinopathy. DME = macular edema diabetic. STDR = sight-threatening DR. * Results with statistically significant differences.

Variable of Interest		Female (*n* = 1545)	Male (*n* = 1975)	*p*-Value
Age (years)		65.4 ± 15.6	64 ± 13.8	0.0419 *
Years from diagnosis		11.1 ± 8.1	9.8 ± 7	<0.0001 *
Mydriasis		97.6%	98%	0.4196
Diabetes type	Type 2	85.8%	90%	=0.0001 *
	Type 1	13.7%	9.6%	=0.0001 *
	Others	0.5%	0.4%	0.6579
DR stage	Mild	8.1%	8.2%	0.9143
	Moderate	10%	9.9%	0.9216
	Severe	0.2%	0.4%	0.2913
	Proliferative	0.2%	0.2%	1
DME		2.1%	2.2%	0.8394
STDR		2.2%	2.4%	0.6952

**Table 3:** Comparison of the common variables between patients with gradable and ungradable images. * Results with statistically significant differences.

Variable of Interest		Gradable	Ungradable	*p*-Value
Gender (F/M)		43.8%/56.2%	44.2%/55.8%	0.8785
Age (years)		63.4 ± 14.6	72.6 ± 11.8	<0.0001 *
Years from diagnosis		10.3 ± 7.3	10.9 ± 9.2	0.1450
Mydriasis		98%	96.6%	0.0678
Diabetes type	Type 2	87.2%	95.6%	<0.0001 *
	Type 1	12.4%	4.2%	<0.0001 *
	Others	0.4%	0%	0.2012

**Table 4:** Number of patients in every pair of diagnostic categories comparing ALG vs. OPH.

	OPH-DR	OPH-NODR	OPH-UNG	Total
ALG-DR	455	379	33	867
ALG-NODR	80	1582	64	1726
ALG-UNG	118	499	310	927
Total	653	2460	407	3520

**Table 5:** Number of patients in every pair of diagnostic categories comparing PCP vs. OPH.

	OPH-DR	OPH-NODR	OPH-UNG	Total
PCP-DR	373	233	25	631
PCP-NODR	214	2005	209	2428
PCP-UNG	66	222	173	461
Total	653	2460	407	3520

**Table 6:** Comparison of the study variables ALG-OPH vs. PCP-OPH. NDME = no diabetic macular edema. DR = diabetic retinopathy. DME = diabetic macular edema. STDR = sight-threatening DR. * Results with statistically significant differences.

Variable of Interest		ALG-OPH (*n* = 2496)		PCP-OPH (*n* = 2825)		*p* Value
Gender (F/M)		42.9%/57.1%		43.6%/56.4%		0.6071
Age (years)		61.4 ± 14.7		62.8 ± 14.6		0.0003 *
Years from diagnosis		10.2 ± 7.3		10.3 ± 7.3		0.6522
Mydriasis		98.3% (2454)		98.6% (2785)		0.3753
Diabetes type	Type 1	14.5% (361)		12.8% (362)		0.0710
	Type 2	85.2% (2126)		86.8% (2451)		0.0928
	Others	0.3% (9)		0.4% (12)		0.5394
NODR		78.6% (1961)		79.2% (2238)		0.5924
DR		21.43% (535)		20.78 (587)		0.5619
DR stage	Mild	10.1% (251)		9.5% (267)		0.4622
	Moderate	10.9% (272)	(NDME 217)	10.8% (305)	(NDME 245)	0.9068
			(DME 55)		(DME 60)	
	Severe	0.3% (7)	(NDME 1)	0.3% (9)	(NDME 2)	1
			(DME 6)		(DME 7)	
	Proliferative	0.2% (5)	(NDME 2)	0.2% (6)	(NDME 2)	1
			(DME 3)		(DME 4)	
DME		2.6% (64)		2.5% (71)		0.8173
STDR		2.7% (67)		2.7% (75)		1

**Table 7:** Unpaired sample. Diagnostic category pairs frequency and diagnostic validity indexes.

Variable of Interest	ALG-OPH (95% CI)	PCP-OPH (95% CI)
True Positive	455	373
False Positive	379	233
False Negative	80	214
True Negative	1582	2005
Prevalence	21.43% (19.80−23.06)	20.78% (19.26−22.29)
Sensitivity	85.05% (81.93−88.16)	63.54% (59.56−67.52)
Specificity	80.67% (78.90−82.45)	89.59% (88.30−90.88)
Positive Predictive Value	54.56% (51.12−58.00)	61.55% 57.60−65.51)
Negative Predictive Value	95.19% (94.13−96.25)	90.36% (89.11−91.61)
Likelihood Ratio +	4.40 (3.99−4.85)	6.10 (5.33−6.99)
Likelihood Ratio -	0.19 (0.15−0.23)	0.41 (0.37−0.45)

**Table 8:** Ranges of likelihood ratio values and their impact on diagnostic accuracy.

Likelihood Ratio +	Likelihood Ratio −	Usefulness
10	<0.1	Highly relevant
5–10	5–10	Good
2–5	2–5	Fair
<2	<2	Poor

**Table 9:** Main descriptors of the sample obtained from de APDR for the study between April 2017 and June 2018. * Mean SD. DME = diabetic macular edema. NDME = macular edema non-diabetic. STDR= sight-threatening DR.

Variable of Interest			Study Sample (*n* = 2335)
Gender (F/M)			42.7%/57.3% (997/1338)
Age (years)			61.1 ± 14.7 *
Years from diagnosis			10.1 ± 7.2 *
Mydriasis (before photograph)			98.8% (2306)
Diabetes type	Type 2		85% (1984)
	Type 1		14.7% (343)
	Others		0.3% (8)
No Diabetic Retinopathy (NODR)			78.9% (1842)
DR prevalence			21.1%
Diabetic retinopathy (DR)	Mild		10.2% (238)
	Moderate	NDME	8.3% (194)
		DME	2.2% (51)
	Severe	NDME	0.04% (1)
		DME	0.2% (5)
	Proliferative	NDME	0.04% (1)
		DME	0.1% (3)
STDR			2.6% (61)

**Table 10:** Paired sample. Diagnostic category pairs frequency and diagnostic validity indexes.

Variable of Interest	ALG-OPH (95% CI)	PCP-OPH (95% CI)
True Positive	418	300
False Positive	351	200
False Negative	75	193
True Negative	1491	1642
Prevalence	21.1% (19.44−22.79)	
Sensibility	84.8% (81.52−88.06)	60.9% (56.44−65.26)
Specificity	80.9% (79.12−82.77)	89.1% (87.69−90.59)
Positive Predictive Value	54.36% (50.77−57.94)	60% (55.61−64.39)
Negative Predictive Value	95.21% (94.12−96.30)	89.48% (88.05−90.91)
Likelihood Ratio +	4.45 (4.02−4.92)	5.60 (4.83−6.50)
Likelihood Ratio −	0.19 (0.15−0.23)	0.44 (0.39−0.49)

## Data Availability

Anonymized data processed in an excel file are available on reasonable request. Restrictions apply to the availability of APDR data. These data are subject to ethical restrictions (Data Protection Act).
